# Nonlinear association between serum insulin, visceral fat area, and kidney function in female with type 2 diabetes: a retrospective study

**DOI:** 10.3389/fendo.2026.1843913

**Published:** 2026-06-01

**Authors:** Yuqin Gan, Mengjie Chen, Zhitao Xiao, Bo Li, Yu Gao, Kaili Dai

**Affiliations:** 1 West China School of Public Health and West China Fourth Hospital, Sichuan University, Chengdu, China; 2 Department of Cardiology, West China Hospital, Sichuan University, Chengdu, China; 3 West China School of Nursing, Sichuan University, Chengdu, China

**Keywords:** Fasting insulin, female patients, nonlinear association, renal function, type 2 diabetes mellitus, visceral fat area

## Abstract

**Objective:**

To investigate the nonlinear associations between fasting insulin (FIns), visceral fat area (VFA), and estimated glomerular filtration rate (eGFR) in female patients with type 2 diabetes mellitus (T2DM), and whether there is a threshold effect.

**Methods:**

This retrospective analysis utilized electronic medical record data from patients with female patients with T2DM who presented to a tertiary hospital in Chengdu, Sichuan Province, between November 1, 2018, and April 30, 2023. Collected data encompassed general demographic characteristics as well as biochemical parameters. The nonlinear associations between FIns, VFA, and eGFR were evaluated using restricted cubic splines and threshold analyses.

**Results:**

A total of 620 female patients with T2DM were initially enrolled; following the exclusion of 25 individuals with incomplete data, 595 female patients were included in the final analysis. After adjusting for confounders such as age and body mass index, significant nonlinear effects were observed in the associations between FIns, VFA, and eGFR. Specifically, there is no threshold between FIns and eGFR; when FIns is <70.28 pmol/L, there is no significant association between FIns and eGFR (β = -0.01, 95% CI: -0.125, 0.105); above 70.28 pmol/L, FIns was negatively correlated with eGFR (β = -0.083, 95% CI: 0.143, 0.023). The threshold for VFA in relation to eGFR was identified as 73.6 cm². When VFA was less than 73.6 cm², a positive correlation with eGFR was observed (β = 0.139, 95% CI: 0.015, 0.262), whereas VFA exceeding this threshold was negatively correlated with eGFR (β = -0.197, 95% CI: -0.33, -0.064).

**Conclusion:**

A nonlinear association was observed between FIns, VFA, and renal function; whereas a distinct clinical threshold was evident in the association between VFA levels and renal function. When FIns levels exceed 70.28 pmol/L, FIns shows a negative correlation with renal function, which may serve as a reference for establishing thresholds in future studies; a VFA of 73.6 cm² represent pivotal inflection points at which renal function transitions from compensatory processes to impairment. This investigation provides novel quantitative evidence for risk stratification of renal function in female patients with T2DM, obesity, and related metabolic disturbances.

## 1 Introduction

Diabetes constitutes a significant global public health challenge, with its prevalence exhibiting a consistent upward trajectory each year. The International Diabetes Federation (IDF) projects that the global prevalence of diabetes will escalate to 12.2% by 2045 (1). Approximately 40% of individuals with diabetes are expected to develop diabetic kidney disease (DKD) (2), and the incidence, prevalence, and mortality associated with DKD continue to rise (3). DKD is typified by a progressive deterioration of renal function and stands as one of the most prevalent and severe chronic complications of diabetes (4). It represents a primary contributor to mortality among individuals with diabetes and is a leading cause of end-stage renal disease. The estimated glomerular filtration rate (eGFR) serves as a comprehensive indicator for evaluating renal function (5). Between 1990 and 2021, the global incidence of DKD secondary to type 2 diabetes mellitus (DKD-T2DM) surged by approximately 85%, with associated mortality more than tripling, thereby positioning DKD among the fastest-growing noncommunicable diseases worldwide (6). Projections indicate that the incidence of DKD will continue to rise through 2036. The escalating burden of DKD not only presents a substantial threat to public health but also adversely affects economic growth and development, highlighting its prioritization in global public health agendas and underscoring the critical need for early screening and intervention (7).

Nevertheless, gender disparities are evident regarding risk factors, screening practices, diagnostic criteria, complications, and therapeutic interventions (8). Following a diabetes diagnosis, women are predisposed to an elevated risk of renal complications, largely attributable to postmenopausal phenotypic alterations, wherein females more frequently exhibit normoalbuminuria concomitant with diminished eGFR (9). Moreover, epidemiological analyses of disease burden in East Asia highlight the imperative for gender-specific preventive strategies (10). Accordingly, this study aims to elucidate the determinants of renal function among female patients with T2DM, thereby furnishing a theoretical foundation for the development of more precise therapeutic approaches and the advancement of patient health outcomes.

T2DM is typified by progressive dysfunction of pancreatic β-cells, culminating in attenuated insulin secretion, a decline in β-cell mass (11), and the development of insulin resistance within peripheral target tissues. These pathological mechanisms underpin the gradual deterioration in glycemic control and the escalating reliance on exogenous insulin therapy (12), while concurrently predisposing patients to a spectrum of both chronic and acute complications (11). Islet cells are a vital endocrine organ that secretes hormones regulating blood glucose homeostasis (13), glucose metabolism, and appetite; any dysfunction in these cells can lead to disturbances in glucose metabolism and trigger diabetes (14). Beyond its primary role in glycemic regulation, insulin exerts direct vascular bioactivity (15). Insulin receptors are ubiquitously expressed across the renal nephron, encompassing the glomerulus and renal tubules, where they modulate a range of critical physiological processes. Aberrations in insulin secretion, irrespective of excess or deficiency, may elicit renal injury through direct or indirect mechanisms, including alterations in vascular tone and the promotion of mesangial cell proliferation, thereby contributing to the pathogenesis of diabetic nephropathy.

Obesity is recognized as a salient contributory factor to hyperinsulinemia, thereby exacerbating the risk of diabetes mellitus (16). While Body Mass Index (BMI) is commonly utilized as a surrogate indicator for adiposity, it is inherently limited in its inability to discriminate among fat depots or accurately reflect fat distribution. Moreover, the metabolic burden imposed by obesity constitutes a principal driver in the development of DKD. Although BMI has traditionally served as a metric for assessing renal metabolic risk, its utility is compromised by its failure to account for body composition and the topographical distribution of adipose tissue. In contrast, the quantification of visceral adipose tissue (VAT) offers greater clinical relevance by capturing the anatomical localization of fat accumulation (17). Notably, in patients with T2DM who possess a normative BMI, an elevated visceral fat area (VFA) independently confers an increased risk for diabetic nephropathy (18). Recent research underscores that the heterogeneity of fat distribution serves as a more robust predictor of metabolic risk than aggregate fat mass (18–20); specifically, visceral adiposity, as opposed to subcutaneous fat, is characterized by augmented lipolytic activity and the enhanced release of free fatty acids and pro-inflammatory cytokines (21). These factors directly precipitate insulin resistance, oxidative stress, and chronic low-grade inflammation, thereby perturbing glucose and lipid metabolism and indirectly compromising renal function. Empirical evidence indicates that individuals with a high VFA bear a 2.47-fold greater risk of developing diabetic nephropathy relative to those with lower visceral fat deposits (22). This heightened risk is chiefly attributed to the “spatial proximity” of visceral fat to the kidneys, particularly perirenal fat, which, as a form of ectopic adiposity, may inflict direct renal injury through the secretion of inflammatory mediators such as TNF-α and the promotion of lipotoxicity (23, 24). Despite significant gender-based differences in adipose tissue distribution, existing studies predominantly treat gender as a confounding variable and typically utilize linear analytical models, resulting in a paucity of nuanced investigations into sex-specific nonlinear associations.

By employing a retrospective analytical approach, this study investigates the nonlinear associations between renal function, female insulin secretory capacity, and VFA from the dual perspectives of diabetic nephropathy pathogenesis: specifically, host factors—including metabolic burden and compensatory capacity. The objective of this study is to elucidate the presence of a threshold effect of insulin secretion and VFA on renal function in female. This study seeks to establish a novel theoretical framework that will serve as the foundation for the development of gender-specific early screening protocols and targeted intervention strategies for DKD in clinical practice. Ultimately, this work aspires to facilitate a paradigm shift in the prevention and management of DKD, transitioning from traditional “one-size-fits-all” methodologies to a more nuanced, individualized precision medicine approach.

## 2 Manuscript formatting

### 2.1 Research methods

#### 2.1.1 Study design and population

Retrospective data collection was conducted among patients with T2DM at the Metabolic Management Center (MMC) of the Endocrinology Department at a Grade A tertiary hospital in Chengdu. The retrospective period spanned from 2018 to 2023, and a total of 620 female patients with T2DM were included in this study.

Inclusion criteria: ① T2DM in accordance with the American Diabetes Association (ADA) 2026 Standards of Medical Care in Diabetes (25); ② Being 18 years of age or older; ③ Female.

Exclusion criteria: ① Patients with other severe chronic comorbidities (e.g., malignant tumors, liver or kidney disease); ② Patients with missing data; ③ Patients with other endocrine disorders, such as pituitary or adrenal gland diseases.

#### 2.1.2 Data collection

Collect the patient’s general information and relevant laboratory data. General information primarily includes age, gender, height, and weight; the BMI is calculated using the ratio of weight to height. Laboratory data primarily includes uric acid (UA), serum creatinine (SCr), triglycerides (TG), total cholesterol (TC), high-density lipoprotein (HDL), low-density lipoprotein (LDL), glycated hemoglobin (HbA_1c_), and the oral glucose tolerance test (OGTT).

The diagnostic criteria for assessing renal function through measurement of serum creatinine (SCr) are based on the eGFR, the Cockcroft-Gault equation was employed to estimate creatinine clearance (CCr) as follows: CCr (mL/min) = (140 – age) × weight (kg) × 0.85/(72 × SCr (mg/dL)), with SCr(μmol/L) = SCr (mg/dL)×88.4. The reference interval for eGFR was defined as 80–120 mL/min; values below this threshold were classified as indicative of impaired renal function (26, 27).

VFA is measured by the National Standardized MMC at the hospital using the Visceral Fat Detector (HDS-2000 DUALSCAN). The device employs bioelectrical impedance analysis (BIA) to collect electrical signals from the subject’s abdomen. Based on feedback from two distinct electrical currents, it effectively distinguishes and calculates VFA; a VFA value greater than 100 cm² is diagnosed as visceral fat-type obesity.

OGTT: Used to evaluate insulin secretion function in pancreatic beta cells. Blood samples are collected from patients in a fasting state early the next morning after an overnight fast to measure fasting insulin levels. Subsequently, patients ingest 75 g of anhydrous glucose or a steamed bun made from 100 g of standard flour, and blood glucose levels are measured 30 minutes, 1 hour, and 2 hours after glucose ingestion. In this study, the Abbott i2000 chemiluminescent immunoassay analyzer was used to measure insulin levels during the OGTT (chemiluminescence method).

#### 2.1.3 Statistical analysis

Data were exported from the EpiData (Chinese version) management software and analyzed using IBM SPSS 26.0 and R 4.5. The classification criteria of <18.5 kg/m² and ≥24 kg/m² used in this study are those recommended by the Chinese Working Group on Obesity (WGOC) for Asian populations (28). A threshold of 60 years was utilized to define age subgroups in this study, as this demarcation is extensive (29), commonly employed in geriatric research and clinical practice; Moreover, within the realm of endocrinology and diabetes research and accompanying clinical guidelines in China, the age of 60 serves as a standard criterion for distinguishing between “non-elderly” and “elderly” individuals with diabetes (30); the normal range for glycated hemoglobin (HbA_1c_) was set at ≤7.0% (25).

Quantitative data are presented as mean ± standard deviation (x̄±s), while qualitative data are presented as frequency and percentage (%). A nonlinear relationship between insulin secretion function, VFA, and renal function in female patients was analyzed using a multivariate linear regression model with restricted cubic spline fitting, and threshold analysis was performed for each model. All tests were two-sided, *P* < 0.05 was considered statistically significant. Given the non-significance of the likelihood ratio test, the identified inflection point is utilized exclusively for descriptive purposes within this dataset; no generalizations to the broader population are made, nor is the robustness of this threshold further evaluated.

#### 2.1.4 Ethical considerations

The study was approved by the Ethics Committee of First Affiliated Hospital of Chengdu Medical College (approval no. 2021-07), and it was carried out in accordance with the Code of Ethics of the World Medical Association (Declaration of Helsinki).

### 2.2 Result

#### 2.2.1 Participant characteristics

This study included a total of 620 female patients with T2DM. There were 25 cases with missing data, and 595 were female. The mean age was 59.49 ± 12.023 years; mean BMI was 23.85 ± 4.432 kg/m²; mean HbA_1c_ was 9.38 ± 2.568, and 21.18% of participants had HbA_1c_ levels within the normal range; Mean VFA was 63.85 ± 33.283 cm², with 83patients (13.95%) having a VFA ≥ 100 cm²; 266 patients (44.7%) had normal renal function, while 329 (55.3%) had abnormal renal function. See **Table 1** for details.

**Table 1:** Female with T2DM sociodemographic characteristics of the study subjects (N = 595).

Variables	Category	Sample n(%)	Mean ± SD
Age(years)	<45	59(9.92%)	59.49 ± 12.023
	45-60	251(42.18%)	
	>60	258(43.36%)	
BMI(kg/m^2^)	<18.5	35(5.88%)	23.85 ± 4.432
	18.5-23.9	492(82.69%)	
	≥24	68(11.43%)	
VFA(cm^2^)	<100	512(86.05%)	63.85 ± 33.283
	≥100	83(13.95%)	
HbA_1c_(%)	≤7	126(21.18%)	9.38 ± 2.568
	>7	469(78.82%)	
eGFR(ml/min)	80-120	266(44.71%)	89.26 ± 41.817
	<80	329 (55.29%)	

BMI, body mass index; VFA, Visceral Fat Area;HbA_1c_, Glycated hemoglobin;eGFR, Estimated Glomerular Filtration Rate.

#### 2.2.2 The nonlinear relationship between fasting insulin, visceral fat area, and renal function

##### 2.2.2.1 The nonlinear relationship between fasting insulin and renal function

**Figure 1:**
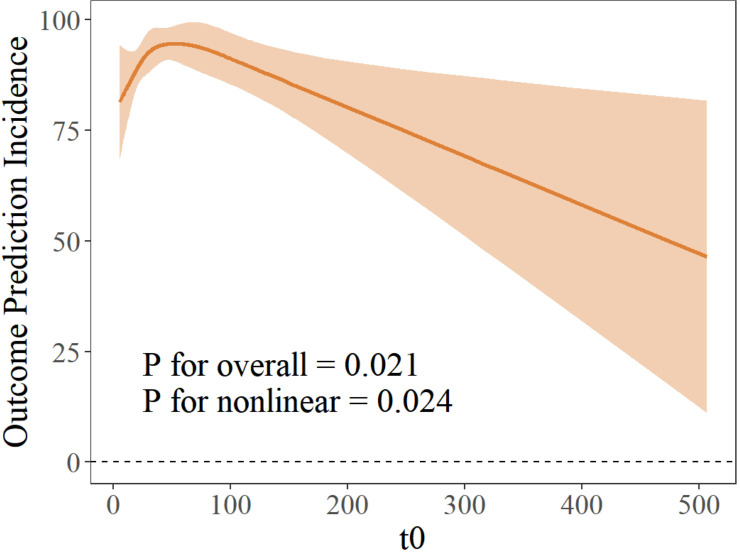
Non-linear relationship between t0 and eGFR: adjusted for age, BMI. t0, Fasting Insulin.

After adjusting for the confounding variables of age and BMI, the model demonstrated a statistically significant nonlinear association between fasting insulin levels and eGFR (*P* < 0.05). The likelihood ratio test yielded a *P*-value of 0.327, suggesting the absence of a threshold effect in the relationship between fasting insulin and eGFR. Specifically, when fasting insulin was below 70.28 pmol/L, no significant association was observed between fasting insulin and eGFR (β = -0.010, 95% CI: -0.125, 0.105). Conversely, when fasting insulin exceeded 70.28 pmol/L, a significant negative correlation was identified (β = -0.083, 95% CI: -0.143, -0.023). See **Table 2**; **Figure 1** for details.

**Table 2:** Threshold analysis of the effect of FIns on eGFR.

Variable	Outcome	The effice sice, 95CI%, *P* value
d1	Model 1 Fitting model by standard linear regression	-0.063(-0.108, -0.019)0.006
d2	Model 2 Fitting model by two-piecewise linear regression	
d3	Inflection point	70.28
d4	<70.28	-0.01(-0.125, 0.105)0.86
d5	>70.28	-0.083(-0.143, -0.023)0.007
d6	P for likelihood ratio test	0.327

Adjusted for age, BMI.

##### 2.2.2.2 Nonlinear relationship between visceral fat area and renal function in female

**Figure 2:**
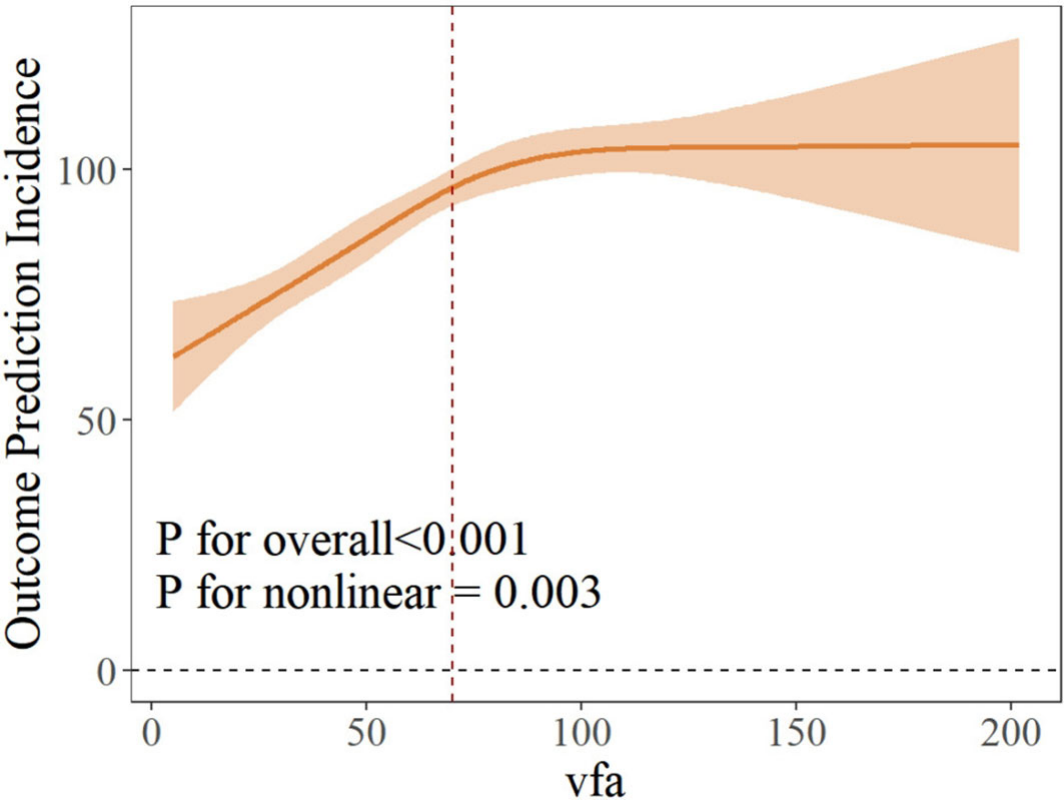
Non-linear relationship between VFA and eGFR. VFA, Visceral Fat Area; adjusted for age, BMI.

The model adjusted for the confounding factors of age and BMI showed that the nonlinear relationship between VFA and eGFR in women was statistically significant (*P* < 0.05), with a threshold of 73.6 cm²; When VFA < 73.6 cm², VFA was positively correlated with eGFR (β = 0.139, 95% CI: 0.015, 0.262), but when VFA > 73.6 cm², VFA was negatively correlated with eGFR (β =-0.197, 95% CI: -0.33, -0.064). See **Table 3**; **Figure 2** for details.

**Table 3:** Threshold analysis of the effect of VFA on eGFR.

Variables	Outcome	The effice sice, 95CI%, *P* value
d1	Model 1 Fitting model by standard linear regression	-0.019(-0.098-0.059)0.628
d2	Model 2 Fitting model by two-piecewise linear regression	
d3	Inflection point	73.6
d4	<73.6	0.139(0.015, 0.262)0.028
d5	>73.6	-0.197(-0.33, -0.064)0.004
d6	P for likelihood ratio test	0.001

Adjusted for age, BMI.

### 2.3 Discussion

The results of this study indicate that there is a nonlinear relationship between fasting insulin (FIns) and eGFR, but no threshold effect. Specifically, when FIns is below 70.28 pmol/L, no significant association with eGFR is observed; conversely, once FIns exceeds this value, a negative correlation emerges between FIns and eGFR. These results are concordant with prior studies demonstrating (31, 32) that elevated serum insulin concentrations are linked to reduced eGFR and compromised renal function. FIns serves as a critical biomarker of glycemic control and pancreatic β-cell functionality; effective regulation of FIns levels can markedly enhance glycemic management and insulin sensitivity in individuals with T2DM (33). This investigation identified 70.28 pmol/L as a clinically meaningful inflection point for fasting serum insulin levels. While this value does not constitute an absolute diagnostic threshold, it serves as a critical reference parameter for subsequent analyses of potential threshold effects. Notably, DeFronzo et al. (34) reported that the corresponding FIns threshold for insulin resistance—assessed by the hyperinsulinemic-euglycemic clamp technique—is approximately 10–12 mU/L. Similarly, Ma et al. (35) identified, in a Chinese cohort, that the optimal HOMA-IR-based cutoff for insulin resistance ranged from 2.1 to 2.5, corresponding to a fasting insulin concentration of approximately 9–11 mU/L, which closely approximates the 70.28 pmol/L (9.8 mU/L) threshold delineated in this study.

FIns is employed as a marker to evaluate pancreatic β-cell secretory capacity and their efficacy in regulating glycemic levels. When FIns levels are below this value, the organism maintains either normative insulin sensitivity or only mild insulin resistance, enabling intrinsic renal regulatory mechanisms to effectively attenuate the influence of insulin oscillations on glomerular filtration dynamics (36, 37). However, once FIns levels exceed this value, a discernible decline in renal function becomes evident; surpassing physiological limits, the deleterious impact of hyperinsulinemia on renal function gradually manifests and predominates. Elevated insulin concentrations may precipitate glomerular hyperfiltration via dilation of afferent arterioles; yet, chronically, this promotes increased glomerular capillary pressure, endothelial injury, and mesangial cell proliferation, cumulatively evolving toward glomerulosclerosis (31). Moreover, heightened insulin levels stimulate the synthesis of endothelin-1 while diminishing nitric oxide bioavailability, thereby disturbing renal vascular tone regulation and facilitating vasoconstriction, inflammatory responses, and aberrant cellular proliferation (38, 39). Superfluous insulin also activates the renin-angiotensin-aldosterone system, augmenting angiotensin II production, which exacerbates glomerular capillary pressure through efferent arteriole constriction and simultaneously enhances sodium reabsorption in the renal tubules, as well as the expression of fibrogenic mediators (40). The results of this study establish a novel theoretical paradigm for elucidating the pathophysiological mechanisms by which insulin influences the initiation and progression of renal disease. Furthermore, these findings furnish a quantitative foundation for clinical risk stratification and the determination of optimal intervention timing. Individuals exhibiting fasting insulin concentrations above 70.28 pmol/L warrant enhanced clinical vigilance, and early, proactive therapeutic strategies may be efficacious in preventing or attenuating the development and progression of chronic kidney disease.

The results of this study delineate a nonlinear association between VFA and eGFR, with a threshold identified at 73.6 cm², corroborating the findings of Miyasato et al. (41).The adoption of a VFA ≥ 100 cm² as a criterion for renal impairment in otherwise healthy individuals offers a novel vantage point on the pathophysiological mechanisms underpinning obesity-related nephropathy. Historically, investigations into the relationship between VFA and renal function have predominantly presumed a linear correlation (42); however, the present findings challenge this paradigm. Specifically, our results indicate a positive correlation between VFA and eGFR when VFA is below 73.6 cm², potentially reflecting a state of glomerular hyperfiltration. In the initial phases of obesity or modest visceral adiposity, elevated metabolic demands instigate increased renal blood flow, thereby eliciting a compensatory augmentation in eGFR (43–45). This hyperfiltration represents an adaptive glomerular response designed to preserve sodium homeostasis amidst heightened tubular reabsorption, yet constitutes a “pseudo” elevation that may heralds early renal decompensation. Notably, this study demonstrates that when VFA surpasses the threshold of 73.6 cm², a negative correlation with eGFR emerges, aligning with existing literature on the concept of “visceral fat toxicity” (41). Once this threshold is surpassed, excessive visceral adiposity may precipitate renal dysfunction through several mechanisms: (1) aberrant accumulation of visceral fat induces ATF6α-mediated downregulation of peroxisome proliferator-activated receptor-α (PPARα) in proximal renal tubular cells, impairing fatty acid β-oxidation, fostering lipid droplet accumulation, and causing mitochondrial dysfunction, augmented endoplasmic reticulum stress, and apoptosis, ultimately advancing tubulointerstitial fibrosis (46); (2) visceral adipose tissue secretes copious pro-inflammatory adipokines, such as TNF-α and IL-6, engendering systemic low-grade inflammation and deleterious structural and functional alterations within the kidneys, while increased circulating free fatty acids exacerbate renal lipotoxicity (47); (3) excessive visceral fat activates the renin-angiotensin-aldosterone system (RAAS) and the sympathetic nervous system, elevating intraglomerular pressure. Collectively, these processes promote the progression of glomerulosclerosis and diminish renal function over time (47, 48).

This study establishes a VFA threshold of 73.6 cm², thereby advancing the previous clinical consensus regarding “visceral fat toxicity”-traditionally defined as VFA ≥ 100 cm² (49)-to an earlier stage and furnishing a more sensitive and quantitative parameter for the early detection and management of CKD. The identification of this threshold suggests that renal pathophysiological alterations may commence prior to the attainment of conventional diagnostic criteria for visceral adiposity. This study determined that a VFA threshold of 73.6 cm² markedly advances the previously established clinical consensus regarding “visceral adiposity toxicity” (VFA ≥ 100 cm²) to an earlier stage, thereby furnishing a theoretical foundation for the proactive prevention and management of CKD. This threshold implies that renal pathophysiological alterations may emerge prior to the attainment of conventionally defined criteria for visceral fat accumulation. Consequently, in clinical practice, this threshold may serve as an early indicator, enabling the prompt identification of individuals at elevated risk and the initiation of targeted interventions to arrest disease progression before irreversible decline in renal function ensues.

### 2.4 Limitations

This investigation was conducted as a single-center, retrospective analysis and, as such, is inherently limited in its capacity to establish causal relationships. Moreover, the generalizability of the findings is constrained to female patients with T2DM, and extrapolation to male subjects or other populations should be approached with caution. The reliance on eGFR derived from serum creatinine may introduce confounding due to variations in muscle mass; thus, future research should employ direct measurement techniques to enhance the precision of eGFR assessment. Additionally, the study did not collect data regarding blood pressure, antidiabetic pharmacotherapy, or diabetes duration, nor did it adjust for the potential confounding effects of these variables on the development of diabetic nephropathy, which may have introduced bias into the observed associations. To substantiate these findings, prospective cohort studies with larger and more diverse samples are warranted.

### 2.5 Conclusion

This study identified a nonlinear association between FIns, VFA, and renal function in female with T2DM. Although a definitive clinical threshold for FIns was not established, levels exceeding 70.28 pmol/L were inversely correlated with renal function, suggesting a potential reference point for threshold determination in future investigations. A VFA of 73.6 cm² was delineated as the threshold at which renal physiology shifts from compensatory hyperfiltration to parenchymal injury. These findings offer substantial theoretical support for the early detection, risk stratification, and intervention of DKD in female, which may contribute to delaying disease onset and progression, enhancing patient outcomes, and optimizing healthcare resource allocation.

## Data Availability

The original contributions presented in the study are included in the article/**Supplementary Material**. Further inquiries can be directed to the corresponding author.
